# A Short Screening Measure of Contextual Vulnerability for Financial Exploitation

**DOI:** 10.1093/geroni/igaa057.1431

**Published:** 2020-12-16

**Authors:** Rebecca Campbell, Peter Lichtenberg

**Affiliations:** Wayne State University, Detroit, Michigan, United States

## Abstract

Financial exploitation (FE) among older adults is an expensive personal and societal problem. Many professionals who work with older adults need fast and reliable measures to detect FE, including: APS workers, financial professionals, and healthcare providers. Very few standardized measures exist to assess an older adult’s risk for FE, and fewer still that do not require extensive training to administer. Lichtenberg et al. (in press) presented the Financial Exploitation Vulnerability Scale (FEVS), which assesses the context (i.e. financial, social, and psychological) in which an older adult is making a financial decision. This 17-item scale differentiates between older adults who’ve experienced FE and those who have not. It has excellent psychometric properties and clinical utility to detect FE beyond demographic and neuropsychological variables. We utilized exploratory factor analysis to examine the factor structure of the FEVS and reduce the number of items. Participant data (n=243) were drawn from two samples: a community-based sample and a group of individuals who were seeking financial coaching services following a financial scam or theft. Examination of the eigenvalues yielded a unidimensional scale. An iterative process was used to remove items that did not load on the single factor or with low corrected item total correlations. The final scale contained nine items and retained its ability to detect financial exploitation (AUC=0.778) and good internal consistency (a=0.845). This short form scale (FEVS-SF) is a brief, standardized screening measure to assess contextual vulnerability for FE that is accessible to professionals who work with older adults.

